# Genomic analysis of a novel *Rhodococcus* (*Prescottella*) *equi* isolate from a bovine host

**DOI:** 10.1007/s00203-019-01695-z

**Published:** 2019-07-13

**Authors:** Megan L. Paterson, Diyanath Ranasinghe, Jochen Blom, Lynn G. Dover, Iain C. Sutcliffe, Bruno Lopes, Vartul Sangal

**Affiliations:** 1 Faculty of Health and Life Sciences, Northumbria University, Newcastle upon Tyne, NE1 8ST UK; 2 Bioinformatics and Systems Biology, Justus-Liebig-Universität, Giessen, Germany; 3 School of Medicine, Medical Sciences and Nutrition, University of Aberdeen, Foresterhill, Aberdeen, AB25 2ZD UK

**Keywords:** *Rhodococcus equi*, Virulence, Pathogenicity island, Bovine, Pneumonia, Plasmid

## Abstract

*Rhodococcus* (*Prescottella*) *equi* causes pneumonia-like infections in foals with high mortality rates and can also infect a number of other animals. *R. equi* is also emerging as an opportunistic human pathogen. In this study, we have sequenced the genome of a novel *R. equi* isolate, B0269, isolated from the faeces of a bovine host. Comparative genomic analyses with seven other published *R. equi* genomes, including those from equine or human sources, revealed a pangenome comprising of 6876 genes with 4141 genes in the core genome. Two hundred and 75 genes were specific to the bovine isolate, mostly encoding hypothetical proteins of unknown function. However, these genes include four copies of *terA* and five copies of *terD* genes that may be involved in responding to chemical stress. Virulence characteristics in *R. equi* are associated with the presence of large plasmids carrying a pathogenicity island, including genes from the *vap* multigene family. A BLAST search of the protein sequences from known virulence-associated plasmids (pVAPA, pVAPB and pVAPN) revealed a similar plasmid backbone on two contigs in bovine isolate B0269; however, no homologues of the main virulence-associated genes, *vapA*, *vapB* or *vapN*, were identified. In summary, this study confirms that *R. equi* genomes are highly conserved and reports the presence of an apparently novel plasmid in the bovine isolate B0269 that needs further characterisation to understand its potential involvement in virulence properties.

**Electronic supplementary material:**

The online version of this article (10.1007/s00203-019-01695-z) contains supplementary material, which is available to authorized users.

## Introduction

*Rhodococcus equi* (“*Rhodococcus hoagii*”/“*Prescottella equi*”), is a Gram-positive, obligate aerobic mycolic-acid containing actinomycete. *R. equi* strains are phylogenomically distinct from other rhodococci and have been proposed to be classified into a novel genus, *Prescottella*, along with *Rhodococcus defluvii* (Jones et al. 2013; Sangal et al. 2015, 2016). The formal nomenclature of this taxon is still waiting clarification (Garrity 2014; Goodfellow et al. 2015). For simplicity, here we refer to the *R. equi*/*R. hoagii*/*P. equi* taxon as *R. equi*.

*Rhodococcus equi* primarily causes pyogranulomas and ulcerative enteritis in young foals (Prescott 1991; Vazquez-Boland et al. 2013) but can also cause sub-maxillary lymphadenitis and respiratory lymph node abscesses in a range of other animals, most notably porcine and bovine species (Vazquez-Boland et al. 2013; Valero-Rello et al. 2015; Ribeiro et al. 2017). It is also notable as an opportunistic human pathogen which is responsible for considerable mortality among immunocompromised patients (Yamshchikov et al. 2010; Giguere et al. 2011). Due to its significant economic impact on the equine breeding industry, recent research has focused on understanding the host–pathogen interaction and the mechanisms of pathogenesis of *R. equi* strains in different hosts (von Bargen and Haas 2009; Vazquez-Boland et al. 2013; Sangal et al. 2014). Notably, the nature of the pathogenicity island carried by the virulence plasmid significantly influences the host association of *R. equi* strains (Valero-Rello et al. 2015; MacArthur et al. 2017; Ribeiro et al. 2017).

In this study, we have sequenced the genome of a novel *R. equi* strain, B0269 that was isolated in 2014 from the faeces of a bovine host in Scotland. Bovine faecal sample (1:10 diluted with sterile saline) was homogenised in BHI enrichment broth and was incubated at ambient temperature for 1 h with occasional agitation. 100 µL of this sample was plated onto blood agar plate (E&O Laboratories, UK) and incubated at 37 °C for 48 h. A single colony was sub-cultured on another blood agar plate and a loopful of the culture was used for genomic DNA extraction using Wizard^®^ Genomic DNA Purification Kit (Promega, USA). The DNA was quantified using the Quant-iT^™^ PicoGreen^™^ dsDNA assay kit (ThermoFisher Scientific, UK). The final concentration of genomic DNA was ~ 30 ng/µl. The genome sequencing was performed on an Illumina Hi-Seq 2000 (Illumina Inc., USA) at the Wellcome Trust Sanger Institute, UK. A total of 4,266,424 paired-end reads with an average read length of 100 bp were assembled into 30 contigs using Velvet (Zerbino and Birney 2008) and were annotated using the RAST pipeline (Aziz et al. 2008; Overbeek et al. 2014; Brettin et al. 2015). The draft genome is 5.7 Mb in size with a 68.4 mol% GC content and 5487 features (5430 coding sequences and 57 tRNA genes) that are comparable to previously sequenced *R. equi* strains (Anastasi et al. 2016; Sangal et al. 2016). The genome sequence of strain B0269 has been deposited at the ENA database under the accession number ERR646794.

For comparative genomic analyses, the publicly available genome sequences of seven *R. equi* strains were obtained from GenBank, i.e., strain 103S, ATCC 33707, C7^T^, N1288, N1295, N1301 and DSM 20295 (Accession numbers: NC_014659, NZ_CM001149, APJC00000000; LRQY00000000; NZ_LRQZ00000000; NZ_LRRA00000000; NZ_LRRF00000000, respectively; Letek et al. 2010; Sangal et al. 2016). These strains were isolated from equine hosts except for strain ATCC 33707, which was isolated from a human, N1288 from a swine host, and N1301 from environment (Qin et al. 2010; Sangal et al. 2014, 2016). Strain DSM 20295 was first described as *Corynebacterium hoagii* in the year 1912 but the source of this strain is unknown (Morse 1912; Kämpfer et al. 2014). To have an equivalence of annotation, these genomes were re-annotated using the RAST pipeline (Aziz et al. 2008; Overbeek et al. 2014; Brettin et al. 2015) and were compared using EDGAR (Blom et al. 2016). Pairwise average amino-acid and nucleotide identities (AAI and ANI) of strain B0269 against 103S, ATCC 33707, C7^T^, N1288, N1295, N1301, and DSM 20295 were calculated using BLAST-based algorithms implemented in EDGAR (Blom et al. 2016).

The pan genome is comprised of 6876 genes, of which 4141 genes belong to the core genome. The core genome is slightly larger than the one calculated by Anastasi et al. (2016), who identified 8174 genes (homologous gene-clusters) in the pan genome including 3858 core genes. Anastasi et al. (2016) used Get_Homologues V2.0 (Contreras-Moreira and Vinuesa 2013) and OrthoMCL algorithm with a 70% sequence identity and 75% coverage in protein homology to define orthologs. In this study, we used EDGAR that applies a more robust approach to determine orthologous genes by calculating Blast Score Ratio Values (Lerat et al. 2003) through an intensively iterative process. The pan genomes are more stringently calculated by pairwise comparison of gene contents of a selected reference using Reciprocal Blast Hits that are filtered according to the orthology criterion based on the Blast Score Ratio Values (Blom et al. 2009, 2016). Therefore, the minor variation in the size of core and pan genomes is likely contributed by the difference in the approach used for calculating the pan genome between these studies.

**Fig. 1:**
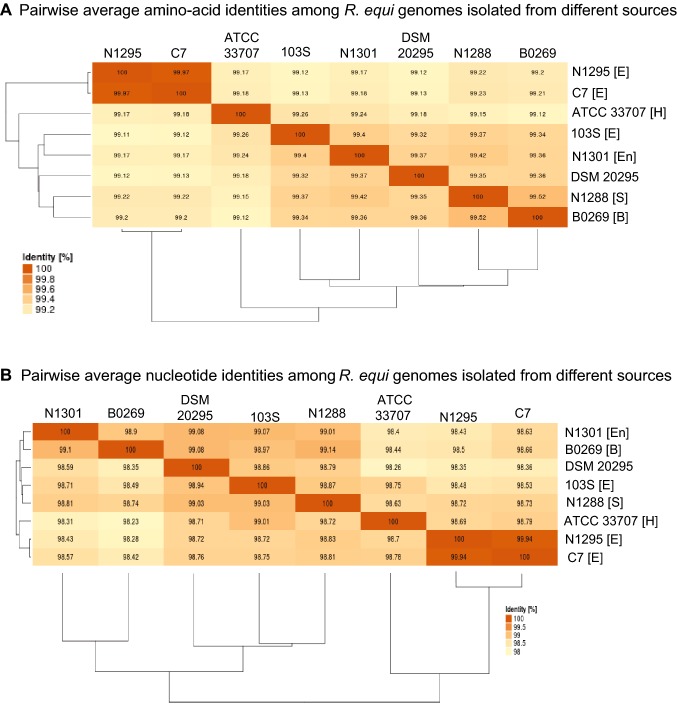
1. Heat maps showing **a** pairwise average amino-acid identities (AAI) and **b** pairwise average nucleotide identities (ANI) among *R. equi* genomes. The source of isolation where known, are mentioned in parentheses next to the strain designations: [B] bovine, [E] equine, [En] environment, [H] human and [S] swine hosts

A maximum-likelihood tree was constructed from the concatenated protein sequence alignment of the core genome using IQ-Tree with 100,000 iterations of ultra-fast bootstrap and 100,000 SH-like approximate likelihood ratio test (Minh et al. 2013; Nguyen et al. 2015). The phylogenetic tree was visualised using the Interactive Tree Of Life (Letunic and Bork 2016), showing a close relatedness of strain B0269 with other *R. equi* isolates (Supplementary Fig. 1). AAI and ANI values of > 99% and > 98%, respectively are consistent with the identification of strain B0269 as *R. equi* and confirm a very high degree of genomic conservation within the species (Fig. 1), as observed previously (Sangal et al. 2015; Anastasi et al. 2016).

Only two hundred and seventy-five genes are found to be specific to *R. equi* strain B0269 that were absent from the other *R. equi* isolates included in this study (Supplementary Table 1). One hundred and thirty-one of these genes encode hypothetical proteins, 9 genes belong to mobile genetic elements (two mobile element proteins and 7 phage-associated genes) while the remaining 135 genes have predictable functions including four copies of *terA* and five copies of *terD* genes. The roles of *ter* gene-clusters remain elusive but they have been implicated to be involved in multiple activities including resistance to tellurite and other xenobiotic compounds, responding to chemical stress and anti-viral defence mechanisms (Anantharaman et al. 2012). Ter family proteins have been found in the closely related species *R. defluvii*, but not in other *R. equi* strains (Sangal et al. 2015). The *ter* operon has also been found to help *Yersinia pestis* survive within macrophages (Ponnusamy and Clinkenbeard 2015) and, therefore, could contribute to the virulence of strain B0269, although we note that strains without *ter* genes survive and multiply within macrophages (Rahman et al. 2005; von Bargen and Haas 2009; Vazquez-Boland et al. 2013). The *ter* region in strain B0269 is larger (~ 7 Kb; reB0269_Peg1159–reB0269_Peg1166) than the one in *R. defluvii* strain Ca11^T^ (~ 4 Kb region; fig|6666666.64062.peg.1365–fig|6666666.64062.peg.1370; Sangal et al. 2015), with average GC content of 64.77 and 65.8 mol%, respectively (Supplementary Fig. 2). Furthermore, additional *ter* genes are present on the same contig in strain B0269 (reB0269_Peg1150–reB0269_Peg1152) and on different contigs in strain Ca11^T^ (fig|6666666.64062.peg.103 and fig|6666666.64062.peg.2208). The discontinuous distribution of the *ter* operon in *R. equi*/*R. defluvii* strains (proposed genus *Prescottella*) suggests this operon may have been acquired by horizontal gene transfer independently by strain B0269.

Three types of virulence plasmids have been identified among *R. equi* isolates (Valero-Rello et al. 2015; MacArthur et al. 2017). Equine and porcine isolates generally harbour circular plasmids, pVAPA and pVAPB, respectively, while a linear pVAPN plasmid has been identified among bovine isolates (Valero-Rello et al. 2015; Ribeiro et al. 2017). *R. equi* strains with any of these plasmids are capable of human infection. Environmental *R. equi* isolates commonly lack the virulence plasmids (Ribeiro et al. 2017). A BLAST search of the protein sequences from pVAPA, pVAPB and pVAPN revealed a plasmid backbone similar to that of pVAPN to be present in strain B0269 with 45 out of 140 genes showing > 50% query coverage (alignment length*100/query length) and > 70% sequence similarities to genes on contigs 2, 9, 11, 12 and 18 (Supplementary Table 2). In contrast, only five pVAPA genes and four pVAPB genes showed > 70% sequence similarities to genes on these contigs. One hundred and seventy-three of the 275 B0269 specific genes are present on these contigs. Sixty-eight of them encoded hypothetical proteins, four genes encoded ABC transporter components for either iron or peptides substrates, and the remaining genes were related to various cellular functions without any obvious association with virulence. This suggests that these proteins may confer novel functionalities to this plasmid type.

Contigs 9 and 18 did not map onto the chromosomal sequence of *R. equi* reference strain 103S when the draft assembly of strain B0269 was aligned using MAUVE (Darling et al. 2010), again consistent with these being plasmid-derived sequences. Only 12% of contig 12 sequence shared similarities with the chromosomal sequence of strain 103S and this contig also likely belongs to the novel plasmid. Ninety-two percent of the contig 2 and 41% of the contig 11 sequences mapped on the chromosome of strain 103S. Only three genes from each of these contigs showed similarity to the plasmid sequences and therefore, the unaligned regions on these contigs may represent genomic islands. In addition, the smaller contigs 23-30 also did not map on the chromosome of 103S and may potentially belong to the plasmid. As noted above, only 45 of the 140 pVAPN genes showed similarities with the proteins in B0269, suggesting that this strain likely possesses a novel plasmid similar to pVAPN but bearing with a distinctive overall gene complement that should be further characterised to understand its potential role in pathogenesis.

## Conclusions

Genomic analyses of eight *R. equi* isolates from diverse sources (environment, equine, bovine and human) confirms that the *R. equi* genome is highly conserved. Bovine strain B0269 possesses multiple copies of *terA* and *terD* genes that are absent from other *R. equi* strains and the functions of these remain to be determined. This strain also apparently carries a novel large plasmid that has a genetic backbone similar to the virulence-associated plasmid pVAPN recovered from other bovine strains; however, further characterisation is needed to understand its potential involvement in virulence properties.

## Electronic supplementary material

Below is the link to the electronic supplementary material.

Supplementary material 1 (PDF 808 kb)

Supplementary material 2 (PDF 1239 kb)

Supplementary material 3 (XLSX 692 kb)

Supplementary material 4 (XLSX 16 kb)
