# Supplementary material for: Genomic analysis of a novel Rhodococcus (Prescottella) equi isolate from a bovine host
Source: Arch Microbiol. 2019 Jul 13;201(9):1317–21. doi: 10.1007/s00203-019-01695-z (PMC6790187; doi:10.1007/s00203-019-01695-z)
Supplement: Supplementary file 1 — Supplementary material 1 (PDF 808 kb) [file 203_2019_1695_MOESM1_ESM.pdf]

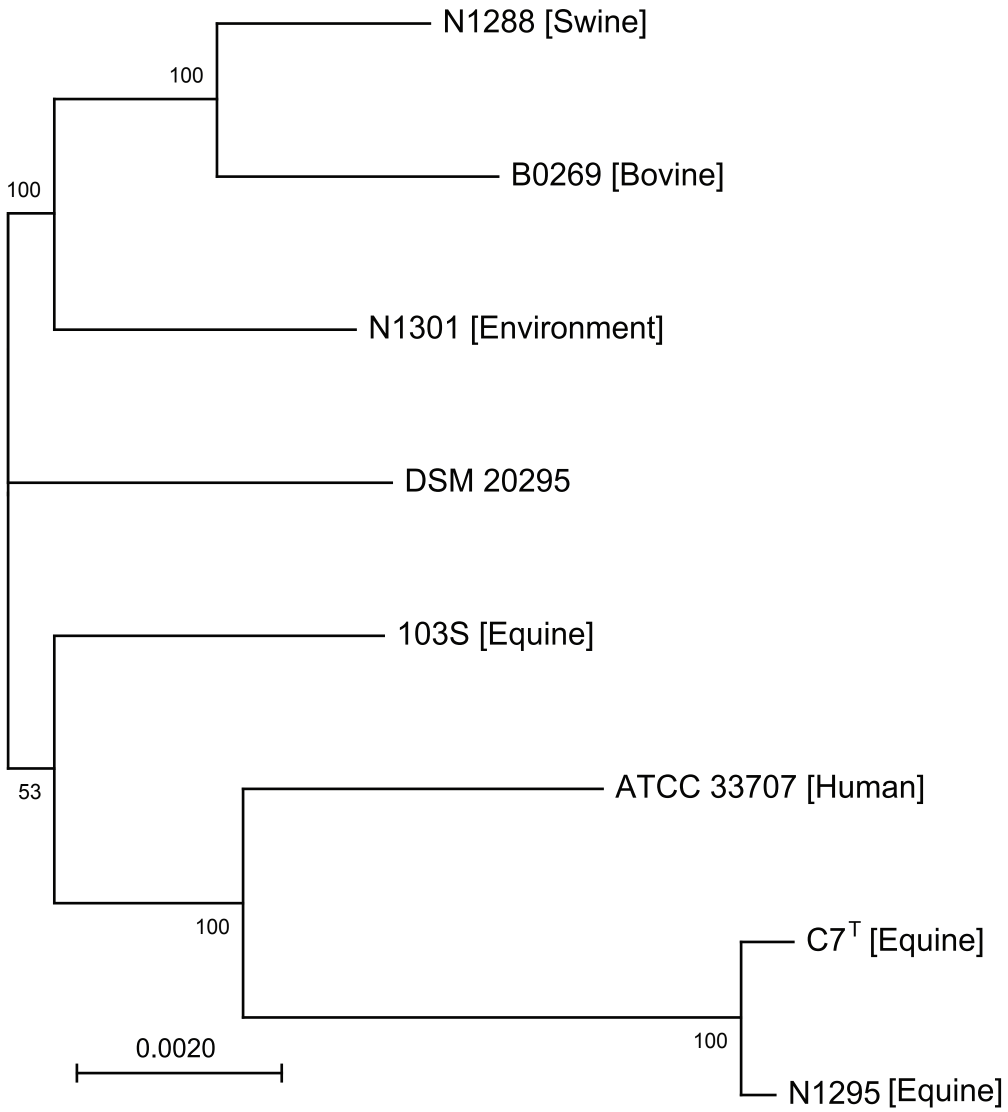

Supplementary Figure 1. A maximum-likelihood tree from the core genome, comprising of 4141 concatenated genes with 1,331,031 amino acid residues per genome, 10,648,248 in total. The tree demonstrates the relatedness of strain B0269 with other *R. equi* isolates. The scale bar represents amino acid substitution per site. The source of isolation for these strains are mentioned in the parentheses next to the strain designations.
