# Supplementary material for: Genomic analysis of a novel Rhodococcus (Prescottella) equi isolate from a bovine host
Source: Arch Microbiol. 2019 Jul 13;201(9):1317–21. doi: 10.1007/s00203-019-01695-z (PMC6790187; doi:10.1007/s00203-019-01695-z)
Supplement: Supplementary file 2 — Supplementary material 2 (PDF 1239 kb) [file 203_2019_1695_MOESM2_ESM.pdf]

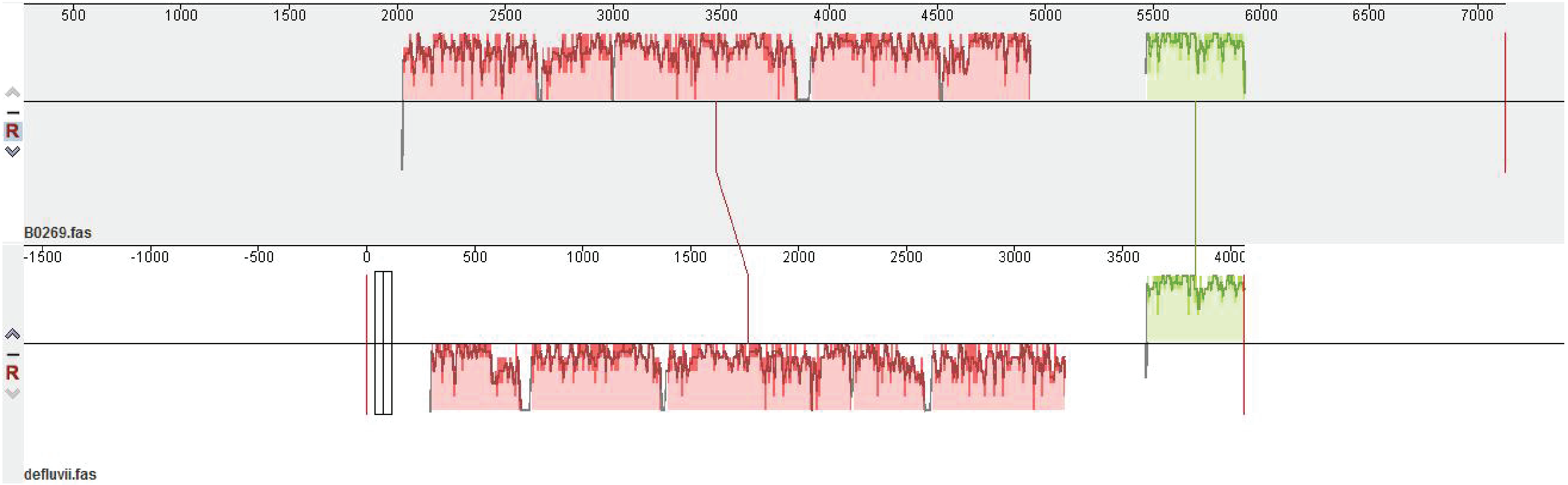

Supplementary Figure 2. A MAUVE alignement of the *ter* operon from *R. equi* strain B0269 (~7 Kb; upper panel) and *R. defluvii* strain Ca11^{T} (~4 Kb; lower panel) highlighting the regions of similarties. Strain B0269 was isolated from a bovine source and Ca11^{T} from wastewater of a bioreactor.
